# Tissue-Based Biomarkers for the Risk Stratification of Men With Clinically Localized Prostate Cancer

**DOI:** 10.3389/fonc.2021.676716

**Published:** 2021-05-28

**Authors:** Spyridon P. Basourakos, Michael Tzeng, Patrick J. Lewicki, Krishnan Patel, Bashir Al Hussein Al Awamlh, Siv Venkat, Jonathan E. Shoag, Michael A. Gorin, Christopher E. Barbieri, Jim C. Hu

**Affiliations:** ^1^ Department of Urology, New York-Presbyterian Hospital, Weill Cornell Medicine, New York, NY, United States; ^2^ Radiation Oncology Branch, National Cancer Institute, Bethesda, MD, United States; ^3^ Department of Urology, University Hospitals Cleveland Medical Center, Case Western Reserve University School of Medicine, Cleveland, OH, United States; ^4^ Department of Urology, University of Pittsburg School of Medicine, Pittsburgh, PA, United States; ^5^ Urology Associates and UPMC Western Maryland, Cumberland, MD, United States

**Keywords:** prostate cancer, tissue biomarker, prognosis, genetic marker, decision making

## Abstract

Risk stratification of men with clinically localized prostate cancer has historically relied on basic clinicopathologic parameters such as prostate specific antigen level, grade group, and clinical stage. However, prostate cancer often behaves in ways that cannot be accurately predicted by these parameters. Thus, recent efforts have focused on developing tissue-based genomic tests that provide greater insights into the risk of a given patient’s disease. Multiple tests are now commercially available and provide additional prognostic information at various stages of the care pathway for prostate cancer. Indeed, early evidence suggests that these assays may have a significant impact on patient and physician decision-making. However, the impact of these tests on oncologic outcomes remains less clear. In this review, we highlight recent advances in the use of tissue-based biomarkers in the treatment of prostate cancer and identify the existing evidence supporting their clinical use.

## Introduction

Until recently, the only available means for risk-stratifying men with clinically localized prostate cancer (PCa) was through the use of clinicopathologic variables such as prostate-specific antigen (PSA) level, histologic grade group, and clinical stage (1, 2). Based on these variables, several nomograms and risk calculators were developed to quantify the risk of disease aggressiveness and assist in patient counseling. The most widely used risk assessment tools include the Partin tables, the Memorial Sloan Kettering Cancer Center (MSKCC) nomogram (3), and the Cancer of the Prostate Risk Assessment (CAPRA) score (4). The Partin tables and MSKCC nomogram are used to predict pathologic tumor and nodal stage following radical prostatectomy (RP). Additionally, the MSKCC nomogram provides information on post-operative cancer-specific and progression-free survival. Likewise, the CAPRA score predicts post-operative pathology including the presences of high-risk features and lymph node involvement as well as recurrence free survival at 3 and 5 years (5–7).

Although these tools offer a reasonable degree of predictive ability, advances in molecular biology have given birth to a variety of urine, blood, and tissue-based tests that provide the physician and patient with additional information about a given patient’s risk for a number of treatment outcomes (8–11). In this review, we aim to discuss tissue-based assays that have become commercially available over the past several years and appraise their utility for treatment planning in men with PCa (**Table 1**).

**Table 1:** Summary of available tissue-based biomarkers and indications.

Test Name	Manufacturer	Genetic Material tested	Endpoint	Test Report	Target Population	Reference
**Repeat Biopsy**						
ConfirmMDx	MDxHealth	Methylation status of 3 genes (GSTP1, RASSF1, APC)	Risk of PCa on repeat biopsy	Likelihood of PCa in %	Men with negative biopsy and considering second one	Stewart et al. (12), Partin et al. (13), Van Neste et al. (14)
**After Biopsy: Active Surveillance vs. Intervention**						
Prolaris Biopsy	Myriad Genetics	Expression levels (RNA) of 31 cell-cycle progression genes	10-year risk of PCa-specific mortality	CCP Score: 0-6	Men with PCa on biopsy	Cuzick et al. (15, 16)
Decipher Biopsy	GenomeDx Biosciences	Expression levels (RNA) of 22 genes (LASP1, IQGAP3, NFIB, S1PR4, THBS2, ANO7, PCDH7, MYBPC1, EPPK1, TSBP, PBX1, NUSAP1, ZWILCH, UBE2C, CAMK2N1, RABGAP1, PCAT-32, GLYATL1P4, PCAT-80, TNFRSF19)	5-year risk metastasis Likelihood of high grade PCa on RP 10-year risk of PCa-specific mortality	GC Score: 0-1.0	Men with localized PCa	Cooperberg et al. (17), Klein et al. (18), Ross et al. (19)
Oncotype DX	Genomic Health	Expression levels (RNA) of 12 genes (AZGP1, KLK2, SRD5A2, FAM13C, FLNC, GSN, TPM2, GSTM2, TPX2, BGN, COL1A1, SFRP4)	Likelihood of GGG 1 or GGG2 on RP Likelihood of organ-confined PCa on RP	GPS Score: 0-100	Men with very low- and low- risk PCa*	Cullen et al. (20), Klein et al. (21)
ProMark	Metamark	Quantitative levels of 8 proteins (DERL1, CUL2, SMAD4, PDSS2, HSPA9, FUS, pS6, YBOX1)	Risk of GGG ≥ 3 or non-organ confined PCa on RP	ProMark Score: 0-100	Men with GGG 1 or 2 on biopsy	Shipitsin et al. (22), Blume-Jensen et al. (23)
PTEN/ TMPRSS2:ERG	Metamark	PTEN deletion and TMPRSS2:ERG fusion		Risk groups	Men with GGG 1 or 2 on biopsy	Yoshimoto et al. (24)
**Management after RP: Further Treatment vs. Observation**						
Prolaris	Myriad Genetics	Expression levels (RNA) of 31 cell-cycle progression genes	10-year risk of BCR	CCP Score: 0-6	Men after RP	Cuzick et al. (25), Cooperberg et al. (17)
Decipher	GenomeDx Biosciences	Expression levels (RNA) of 22 genes	5-year risk of metastasis 10-year risk of PCa specific mortality	GC Score: 0-1.0	Men with high-risk pathology or high-risk clinical features after RP	Karnes et al. (26), Den et al. (27)

PCa, prostate cancer; BCR, biochemical recurrence; CCP, cell cycle progression; GC, genomic classifier; GGG, Gleason grade group; GPS, genomic prostate score; RP, radical prostatectomy.

*based on NCCN risk group.

### Decipher

The Decipher test (Decipher Biosciences, San Diego, CA, USA) uses a microarray platform to measure the expression levels of 22 genes (LASP1, IQGAP3, NFIB, S1PR4, THBS2, ANO7, PCDH7, MYBPC1, EPPK1, TSBP, PBX1, NUSAP1, ZWILCH, UBE2C, CAMK2N1, RABGAP1, PCAT-32, GLYATL1P4, PCAT-80, TNFRSF19) that participate in multiple biologic pathways, such as cell proliferation, differentiation, adhesion and cell cycle progression, and androgen receptor signaling (28). The test requires the extraction of RNA from formalin-fixed paraffin-embedded tissue and a tumor specimen measuring at least 0.5 mm (29). A Decipher Biopsy score is generated when the assay is performed on biopsy tissue, and a Decipher Radical Prostatectomy score is generated when the assay is performed on a RP specimen. Both scores are reported as a number ranging from 0 to 1. A score of 0 to 0.45 is defined as low-risk, 0.46 to 0.6 is average-risk, and above 0.61 is high-risk.

The Decipher Biopsy report provides an assessment of adverse pathology at time of RP, as well as the risk of metastasis and PCa-specific mortality at 5 and 15 years, respectively. The Decipher Radical Prostatectomy report provides similar information with respect to risk of metastasis and prostate-cancer specific mortality, with the goal of guiding decision-making regarding the use of adjuvant radiotherapy, however the clinical utility of this test has never been prospectively validated. Given the recent results of GETUG-AFU 17, RADICALS-RT, and RAVES summarized in the ARTISTIC meta-analysis, which suggest similar outcomes to a strategy of salvage radiotherapy when compared to adjuvant radiotherapy for patients with high-risk histopathologic findings, the utility of genomic classifiers (GCs) may now be somewhat limited in this clinical setting (30–33). Open questions include the clinical benefit of risk stratification with GCs for the selection of adjuvant radiotherapy in select patients with multiple risk factors (34), the possibility of GCs to identify a population unlikely to benefit from salvage radiotherapy (35), and the possible application of GCs in the selection of patients to undergo androgen deprivation therapy as an adjunct to salvage radiotherapy (36).

The expression signature of the genomic classifier that underlies the Decipher test was originally developed using RP specimens from a cohort of men treated at the Mayo Clinic (28). A panel of more than 1.4 million genomic markers, including coding and non-coding RNAs, were compared between 192 men with metastatic PCa and 353 controls. The area-under-curve (AUC) for the genomic classifier was 0.90 in the original cohort and was additionally validated in a second cohort of 186 patients where the AUC was 0.75. In this study, the genomic classifier was the strongest predictor of metastasis in a multivariable analysis (P < 0.001). After the initial validation, further studies expanded its use to predict metastasis (18, 19, 37) and prostate-cancer specific survival after RP (26, 38).

Most of the data to support Decipher Biopsy come from studies done on RP specimens. However, in 2016, Knudsen et al. demonstrated the applicability of the Decipher test in tissue derived from biopsy specimens (39). The authors were able to show that almost 95% of the transcriptomic information extracted from RP specimens could also be derived from biopsy tissue with high correlation (r = 0.96) (39). Several subsequent studies demonstrated the clinical efficacy of the Decipher Biopsy test (40–42). For example, Klein et al. found that Decipher score from prostate biopsy specimens was a significant predictor of metastasis within 10 years after RP with an AUC of 0.8 (43).

Multiple studies have evaluated the role of Decipher testing in clinical decision making (44–46). For example, PRO-ACT was a prospective study that evaluated the treatment decisions of 15 community urologists before and after exposure to the Decipher test results (47). In total, 60% of patients with high-risk disease were reclassified as low risk based on the results of this test and the decision to proceed with adjuvant radiation was changed in 30% of cases. Additionally, 42% of patients who were initially recommended to undergo adjuvant therapy were subsequently reassigned to observation following Decipher testing. In this study, the use of Decipher significantly changed urologists’ adjuvant treatment recommendations for men who were at high risk of metastasis post-prostatectomy (P < 0.001) (47). PRO-IMPACT demonstrated similar results (45). This was a prospective study evaluating the impact of Decipher testing on decision making for adjuvant and salvage radiation therapy in 265 post-prostatectomy patients found to have either adverse pathology or a rising PSA. Prior to Decipher testing, observation was recommended for 89% of patients considering adjuvant radiation and 58% of patients considering salvage treatment. After Decipher testing, 18% of treatment recommendations changed in the adjuvant radiation arm and 32% in the salvage arm. In both groups, the Decipher test was associated with significant decrease in decisional conflict for both physicians and patients (P < 0.001). Finally, the role of Decipher has been evaluated as a guide for androgen deprivation therapy after adjuvant or salvage radiotherapy post-prostatectomy (48). In this setting, a low Decipher score predicts a more favorable prognosis and may change treatment intensification strategies (40). Recently, Jairath et al. performed a systematic review on the available evidence on Decipher and its role on PCa management (40). The authors concluded that in multiple studies Decipher was an independent prognostic factor for adverse pathology, biochemical failure, metastasis, and cancer-specific and overall survival. Decipher’s utility seems to be more important for intermediate-risk PCa as well as post-prostatectomy decision-making.

According to the guidelines from the National Comprehensive Cancer Network (NCCN), the Decipher test may be offered to men with very-low, low- and intermediate-risk PCa on biopsy and a life expectancy of at least 10 years. The goal of the test in this context is to aid in the selection of candidates for active surveillance. Post-prostatectomy, the Decipher test may be offered to men with pT2 disease and positive surgical margins or any pT3 disease to aid in the decision whether to undergo adjuvant radiation therapy (49).

### Prolaris

The Prolaris Molecular Score (Myriad Genetics, Salt Lake City, UT, USA) assay measures the expression of 31 cell cycle progression (CCP) genes related to cancer proliferation and can be performed on either a biopsy or RP specimen (25). The CCP score ranges from 0 to 10, with a high score indicating a more aggressive cancer and correlating with a high risk for disease progression (15, 16). Each 1-unit increase reflects a doubling in gene expression level, suggesting a more aggressive tumor. The CCP score has been used for men with newly diagnosed PCa (Prolaris biopsy test) as well as men who have already undergone prostatectomy (Prolaris post-prostatectomy test). The Prolaris biopsy test reports the risk of 10-year PCa-specific mortality and 10-year metastasis with definitive treatment, whereas the Prolaris post-prostatectomy test reports the risk of 10-year biochemical recurrence.

The Prolaris assay is comprised by an index of 31 genes which were felt most reliably to model the entirety of the identified set of CCP genes. The predictive utility of this gene signature was first reported in a retrospective study which showed a significant correlation between the CCP score and clinical outcomes in two separate cohorts, the first comprised of 366 patients who had undergone and the second 337 men with localized PCa diagnosed by a transurethral resection who were managed conservatively. The CCP score was associated with risk of biochemical recurrence (HR 1.77, 95%CI 1.40–2.22, P < 0.001) in the prostatectomy cohort and PCa specific mortality (HR 2.57, 95%CI 1.93–3.43, P < 0.001) in the conservatively managed cohort (16).

The predictive utility of the CCP score was first defined in a 2011 report in which the authors used two different patient cohorts for validation (16). The first cohort had 366 patients who had undergone RP, and the second cohort had 337 men with clinically localized PCa diagnosed by a transurethral resection (TURP) who were managed conservatively. In this study, the CCP score was associated with risk of biochemical recurrence (HR 1.77, 95%CI 1.40–2.22, P < 0.001) in the prostatectomy cohort and PCa specific mortality (HR 2.57, 95%CI 1.93–3.43, P < 0.001) in the conservatively managed cohort (16).

The Prolaris post-prostatectomy test was subsequently validated on another independent cohort of 413 men by Cooperberg and co-workers (17). In this study the authors demonstrated that when controlling for clinicopathologic factors, CCP score was a strong predictor of biochemical recurrence with each increase in score (HR 2.1, 95%CI 1.6 to 2.9, P < 0.001) (17). Based on this finding, Prolaris may be used to select men who are candidates for post-prostatectomy adjuvant therapy. A later study by Koch et al. showed that men with increased CCP score who had biochemical recurrence after RP had increased risk of systematic disease, suggesting that this patient population could benefit from earlier adjuvant therapy (10, 50).

The Prolaris biopsy test can facilitate decision-making process for men considering active surveillance *versus* localized treatment (surgery or radiation). Bishoff et al. evaluated the CCP score in prostate biopsy specimens of 582 men who underwent radical prostatectomy and demonstrated that increased biopsy CCP score was associated with biochemical recurrence (HR per score unit 1.47, 95%CI 1.23–1.76, P < 0.001) and metastatic progression (HR per score unit 4.19, 95%CI 2.08–8.45, P < 0.001) (51). In 2015, Cuzick et al. demonstrated in a study of 585 men undergoing active surveillance that biopsy CCP score is an independent predictor of prostate-cancer specific mortality (HR per score unit 1.76, 95%CI 1.44–2.14, P < 0.001) after adjusting for Gleason score, PSA, extent of disease, and clinical stage (15).

The Prolaris biopsy test also provides a 10-year PCa specific mortality risk upon combining the patient’s PSA, clinical stage, % of positive cores, biopsy grade group, and AUA risk group (52). The PROCEDE-1000, a large, prospective registry with almost 1,600 participants, showed that the CCP score resulted in a change in treatment for 47.8% of patients (53). More specifically, treatment was deescalated in 75% of cases and escalated in 25% of cases. In spite of CCP score’s use as a means to help physicians and patients reach personalized treatment decisions, no prospective data have shown clinical superiority of the decisions that the test informs.

According to the NCCN guidelines (49), Prolaris biopsy test may be recommended to men with very-low, low-, and favorable intermediate-risk PCa on biopsy and a life expectancy of at least 10 years.

### PTEN/TMPRSS2:ERG

The PTEN/TMPRSS2:ERG (Metamark, Cambridge, MA, USA) assay detects the presence of both *PTEN* and the fusion *TMPRSS2:ERG* genes in biopsy specimens. Deletion of *PTEN* and/or presence of *TMPRSS2:ERG* indicates more aggressive PCa (54).

*PTEN* is a tumor suppressor gene that helps regulate cell division by modifying other proteins and lipids *via* phosphatase action. *PTEN* loss results in deactivation of the PI3K signaling pathway which controls cell growth and proliferation (55). Loss of *PTEN* in PCa has been associated with high cancer grade group, tumor progression and poor outcomes (56, 57). Yoshimoto et al. demonstrated that men with homozygous *PTEN* deletion are more likely to develop late biochemical recurrence (P = 0.005) (24).

TMPRSS2:ERG fusion gene is a common chromosomal rearrangement in PCa. While TMPRSS2:ERG fusion gene has not been found to be a strong predictor of biochemical recurrence and PCa-specific mortality, its presence is associated with higher T-stage and higher risk of metastasis (58, 59). Ahearn et al. showed that loss of PTEN in the presence of TMPRSS2:ERG fusion is independently associated with PCa progression (60). Heterozygous or homozygous *PTEN* loss was associated with PCa specific mortality in the absence of ERG fusion. However, this association was not seen in patients with a loss of PTEN in the presence of ERG fusion. Therefore, the presence of TMPRSS2:ERG fusion may modulate the effects of PTEN loss on the disease biology (10, 60).

The impact of the PTEN/TMPRSS2:ERG tissue assay on the decision making process regarding therapy has not been studied yet. However, the MyProstateScore (LynxDx, Inc., Ann Arbor, Michigan, USA) test is a recent advancement that uses urinary TMPRSS2:ERG, urinary PCa antigen 3, and serum PSA to rule out grade group ≥2 cancer in biopsy naïve men (61). Currently, PTEN/TMPRSS2:ERG is available as a standalone test for men with atypical pathology, high-grade prostatic *in situ* neoplasia and those with grade group 1 or 2 PCa to provide risk stratification (10). However, both PTEN mutations and TMPRSS2:ERG fusions are regularly tested as part of commercially available next generation sequencing (NGS) panels such as FoundationOne CDx (62). The latter is the first FDA-approved tissue-based broad companion diagnostic (CDx) that is clinically and analytically validated for all solid tumors.

The PTEN/TMPRSS2:ERG assay is not recommended as standalone test for routine use in the most recent NCCN guidelines. However, germline genetic testing is now supported by NCCN guidelines for all men with high-risk, very-high-risk, regional or metastatic PCa as well as men with PCa who have Ashkenazi Jewish ancestry or family history of high-risk germline mutations (*e.g.* BRCA1/2, Lynch Syndrome). Furthermore, men with PCa and positive family history for cancer (brother or father or multiple family members with PCa under the age of 60 or more than three cancers on the same side of family) should also undergo germline genetic testing (49).

### Oncotype DX

Oncotype DX (Genomic Health, Redwood City, CA, USA) is an assay that utilizes reverse transcriptase-PCR to measure the expression levels of 12 cancer genes and five housekeeping genes. The 12 cancer genes are components of four major cellular pathways: proliferation (TPX2), androgen receptor pathway (AZGP1, KLK2, SRD5A2, FAM13C) cellular organization (FLNC, GSN, TPM2, GSTM2) and stromal response (BGN, COL1A1, SFRP4). The combination of these genes is used to calculate the Genomic Prostate Score (GPS), which ranges from 0 to 100. GPS correlates with the probability of adverse pathology, such as primary grade group and/or non-organ confined disease at the time of prostatectomy (63).

Initially introduced for breast (64) and colon cancer (65), the Oncotype Dx test was approved for use in PCa in 2013. Klein et al. validated Oncotype DX using three cohorts of patients: prostatectomy discovery cohort, prostate biopsy cohort, and an independent prostate biopsy validation cohort (21). The authors first explored 732 candidate genes in the prostatectomy discovery cohort and identified 288 genes predictive of clinical recurrence and 198 genes predictive of aggressive disease after adjustment for PSA, grade group, and clinical stage. These genes were then evaluated in a prostate biopsy cohort to identify a subset that is associated with adverse pathology at prostatectomy. This analysis led to the development of current test’s 17 gene panel which was independently validated in an unrelated 395 patients with available prostate biopsy and prostatectomy pathology. Notably, this cohort included only men with low-volume intermediate-risk PCa. GPS predicted high-grade and high-stage disease at RP. Another study by Cullen et al. showed that GPS score can predict adverse pathology at prostatectomy but also eventual post-treatment biochemical recurrence (HR 2.73, 95%CI 1.84–3.96, P < 0.001 per 20 GPS units increase) (20).

Regarding the role of Oncotype DX in clinical decision making, Badani et al. performed a prospective study in 158 men with very low to low-intermediate risk PCa to assess the impact of incorporating Oncotype DX on treatment recommendations (66). The authors found that the use of Oncotype DX resulted in an 18% overall change in treatment recommendation. More specifically, active surveillance increased from 41 to 51%, prostatectomy decreased from 21 to 19% and radiation therapy decreased by 33%.

Furthermore, while the predictive utility of the Oncotype DX score to prognosticate adverse pathologic or clinical outcomes has been well validated, its prospective utility as a decision aid to modify treatment recommendations still requires validation in PCa. However, based on advances in the field of breast cancer, it is hopeful that this translation will be fruitful. While the Oncotype DX score for breast cancer was initially validated in the NSABP B20 cohort as a predictive marker for distant metastases (67), a more recent prospective trial, TAILORx, has demonstrated prospective utility as a decision aid to identify a subgroup of women with higher risk, early stage node-negative disease in whom omission of chemotherapy is appropriate (68–70).

According to the NCCN guidelines, Oncotype DX may be offered to men with very-low, low- or favorable intermediate-risk PCa on biopsy and a life expectancy of at least 10 years (49).

### ConfirmMDx

ConfirmMDx for PCa (MDxHealth, Inc., Irvine, CA, USA) is a tissue-based assay that can be used for risk stratification of men with negative prior prostate biopsies. This test involves quantifying the methylation of promoter regions of three tumor suppressor genes (*RASSF1*, *GSTP1*, and *APC*) in benign prostate biopsy tissue (14, 71). When the CpG islands expand in the promoter regions of these genes, there is an increased risk for PCa development. The concept behind this test is that the normal prostatic tissue surrounding an area of adenocarcinoma will undergo epigenetic changes (72).

The two major studies that validate the use of ConfirmMDx are the Methylation Analysis to Locate Occult Cancer (MATLOC) and Detection of Cancer Using Methylated Events in Negative Tissue (DOCUMENT) (12, 13). The MATLOC study demonstrated that ConfirmMDx has sensitivity and specificity of 68 and 64%, respectively, for identifying occult PCa, defined as having a negative biopsy followed by a positive biopsy within 30 months. Furthermore, it showed that ConfirmMDx decreased the number of unnecessary prostate biopsies by up to 64% (12). The DOCUMENT study showed that ConfirmMDx is an independent predictor for PCa when compared to other clinicopathologic parameters and has a negative predictive value of almost 90% (13). Furthermore, Van Neste et al. concluded that men with low DNA-methylation levels in benign biopsies had a negative predictive value of 96% for high-grade cancer (73). The most recent clinical trial on ConfirmMDx is PASCUAL (NCT02250313), which has yet to be reported after termination in 2018. Nevertheless, it’s important to note that these trials were performed prior to the adoption of prostate MRI in the diagnostic algorithm of PCa. Therefore, the role of ConfirmMDx should be reevaluated in the era of MRI-targeted prostate biopsies.

Regarding the role of ConfirmMDx in clinical decision making, Wonju et al. found that only 4.4% of men with negative ConfirmMDx had repeat biopsy, compared to a 43% repeat biopsy rate in the PLCO trial (74). In this study, all the repeat biopsies of patients with negative ConfirmMDx were also negative. Moreover, Van Neste et al. demonstrated that if a probability threshold of 15% is applied, then 30 unnecessary repeat biopsies could be avoided per 100 patients (9, 73).

CONFIRMMDX has not been incorporated in the most recent NCCN guidelines.

### ProMark

The ProMark test (Metamark, Cambridge, MA, USA) is a protein-based assay that measures the levels of eight proteins (DERL1, CUL2, SMAD4, PDSS2, HSPA9, FUS, pS6, and YBOX1) in a prostate biopsy specimen through quantitative immunofluorescence. These proteins participate in cell signaling, stress response and cell proliferation (9). The concept behind evaluating protein levels is based on the significant intratumoral heterogeneity that characterizes PCa. Thus, a protein-based panel aims to provide information derived from the most aggressive cells that might exist in a tumor.

ProMark reports a score from 0 to 1 that reflects the probability of Gleason score ≥4 + 3 disease or non-organ confined disease on RP. The test is meant to be used by men who are NCCN very-low or low-risk and considering active surveillance.

Initially, Shipitsin et al. reported 12 protein biomarkers that predicted PCa aggressiveness and lethal outcome in both high- and low-Gleason areas (22). In 2015, Blume-Jensen et al. used eight of the 12 protein biomarkers in 381 matched prostate biopsy and prostatectomy specimens to validate the eight-biomarker assay as a predictor of prostate pathology (23). More specifically, they showed that a “favorable” score of ≤0.33 is predictive of favorable pathology in 95% of very low-risk and 81.5% of low-risk NCCN patients. The predictive value for non-favorable pathology was 76.9% at biomarker risk scores >0.8 across all risk groups. The authors also performed a validation study in 276 cases and were able to show that the eight-protein biomarker separates favorable from non-favorable disease as well as Gleason score 6 disease *versus* non-Gleason score 6 disease (AUC 0.68 and 0.65, respectively).

According to the NCCN guidelines, ProMark is recommended for men with very-low or low-risk PCa on biopsy and a life expectancy of at least 10 years (49).

### Limitations of Tissue-Based Biomarkers

Tissue biomarkers for PCa need to be used within the context of their limitations. First, the majority of the tissue-based biomarkers have been validated in cohorts primarily consisting of White Caucasian men. However, there are multiple reports demonstrating that the aggressiveness of PCa differs among races (75–78). This stands true especially for African American men in whom there is a higher incidence and mortality secondary to PCa (79). While there is emerging data suggesting that the mortality difference between African American and White males may be a product of unequal access to care rather than genetics, this is still an area of active research. Therefore, the use of genetic risk classifiers in African American men likely requires further validation. Second, most of the tissue-based biomarkers have inconsistent coverage from insurances in the United States. Thus, the financial burden may preclude their use for certain patient populations. Third, there is lack of data regarding cost-effectiveness. Lobo et al. demonstrated that a Decipher-based care model could lead to cost savings of approximately 25% without any significant change in life expectancy (80). However, the literature lacks similar reports on the other available tissue biomarkers Fourth, the heterogeneity and multifocality of primary PCa should not be ignored. As demonstrated by Salami et al. gene expression assays performed on low-grade PCa biopsy tissue may not provide meaningful information on the presence of coexisting unsampled aggressive disease (81). More specifically, multifocal, low-grade and high-grade PCa foci can exhibit distinct prognostic expression signatures within the same case. Recent studies have also characterized significant changes to the genomic classifier scores in some patients depending on the biopsy core or area of the prostatectomy specimen analyzed suggesting the challenges of genomic risk classification in tumors with clonal and genomic heterogeneity (81–83). Fifth, many of the tissue biomarker related studies were performed in the pre-MRI era. Thus, it remains unclear if biomarkers provide clinically useful information in the management of localized PCa beyond MRI-guided interventions and treatment decisions. Furthermore, given the lack of head-to-head comparative studies, there is no level 1 evidence to establish the superiority of a single tissue biomarker over another and thus the choice of biomarker falls to the patient or clinician and may be somewhat dependent on financial factors (49). Therefore, there is no tissue-based biomarker that is considered “better” than others and it is each individual clinician’s decision after discussion with his patients which to choose. Moreover, as recommended by the American Society of Clinical Oncology and the European Association of Urology, while tissue-based biomarkers could aid in the decision-making process for some men with PCa, they should not be offered routinely to everyone (84, 85). Finally, it needs to be highlighted that the literature lacks prospective studies supporting the role of tissue biomarkers as means to guide specific therapies (e.g. salvage or adjuvant treatment) and impact PCa-specific outcomes. Trials similar to the TAILORx trial in breast cancer need to be performed for PCa tissue biomarkers to evaluate their impact in disease specific outcomes.

### Conclusions and Future Directions

A multitude of tissue-based genomic tests have emerged in recent years, providing prognostic information beyond that of standard clinicopathologic variables. These assays are available at various stages in the care pathway of PCa and offer insight into the risk of high-grade disease, rate of metastasis, and cancer-specific survival. However, many challenges lie ahead. To date, Decipher and Prolaris have the most supporting data available but, again, neither has been proven superior in comparative studies. Although some tests have demonstrated an ability to significantly impact management—guiding the pursuit of active surveillance, definitive therapy, and adjuvant radiation post-prostatectomy, there is lack of prospective studies supporting their impact on disease specific outcomes. Given the multiple commercial options for tissue-based biomarkers, it is likely that market forces including industrial investments in direct-to-consumer and direct-to-provider advertising will be major drivers of assay uptake and usage in clinical practice. Representation in national guidelines has already begun and will likely continue to grow as more genomic markers of PCa are discovered. However, incorporation in the daily clinical practice and insurance coverage still constitute areas that more work needs to be done so physicians and patients can benefit. Such assays may soon claim a central role in the management of men with PCa and deserve recognition as facilitators of an individualized approach to patient care.

## Author Contributions

All authors contributed to data gathering and manuscript drafting and review. All authors contributed to the article and approved the submitted version.

## Funding

JH receives research support from the Frederick J. and Theresa Dow Wallace Fund of the New York Community Trust. JH also receives salary support from NIH R01 CA241758, PCORI CER-2019C1-15682 and CER-2019C2-17372. JS is supported by the Frederick J. and Theresa Dow Foundation of the New York Community Trust and a Damon Runyon Cancer Research Foundation Physician Scientist Training Award.

## Conflict of Interest

The authors declare that the research was conducted in the absence of any commercial or financial relationships that could be construed as a potential conflict of interest.
